# Alternative treatments for oral bisphosphonate-related osteonecrosis of the jaws: A pilot study comparing fibrin rich in growth factors and teriparatide

**DOI:** 10.4317/medoral.19458

**Published:** 2014-03-08

**Authors:** Alejandro Pelaz, Luis Junquera, Lorena Gallego, Luis García-Consuegra, Sonsoles Junquera, Carlos Gómez

**Affiliations:** 1 MD, DDS, PhD. Department of Maxillofacial Surgery. Leon University Hospital, Spain; 2 MD, DDS, PhD. Department of Maxillofacial Surgery. University of Oviedo. Central University Hospital, Spain; 3 MD, DDS, PhD. Department of Maxillofacial Surgery. Cabueñes Hospital, Gijón, Spain; 4 Medical student. University of Santiago de Compostela, Spain; 5 MD, PhD. Unit of Bone and Mineral Metabolism. Central University Hospital of Oviedo, Spain

## Abstract

Objectives: The aim of this study is to describe and compare the evolution of recurrent bisphosphonate-related osteonecrosis of the jaws (BRONJ) in patients treated with plasma rich in growth factors or teriparatide. Material and Methods: Two different types of treatments were applied in patients diagnosed of recurrent BRONJ in a referral hospital for 1.100.000 inhabitants. In the group A, plasma rich in growth factors was applied during the surgery. In the group B, the treatment consisted in the subcutaneous administration of teriparatide. All the cases of BRONJ should meet the following conditions: recurrent BRONJ, impossibility of surgery in stage 3 Ruggiero classification and absence of diagnosed neoplastic disease. Clinical and radiographic evolution of the patients from both groups was observed. Results: Nine patients were included, 5 in group A and 4 in group B. All the patients were women on oral bis-phosphonate therapy for primary osteoporosis (5 patients) or osteoporosis-related to the use of corticosteroids (4 patients). Alendronate was the most common oral bisphosphonate associated with BRONJ in our study (four patients in group A and two in group B). The mean age was 72,8 years in the group A and 73,5 years in the group B. All the patients from group A showed a complete resolution of their BRONJ. Only one patient in the group B showed the same evolution. Conclusions: In our series, the plasma rich in growth factors showed better results than the teriparatide in the treatment of recurrent BRONJ.

** Key words:**Osteonecrosis, oral bisphosphonate, treatment, teriparatide, plasma rich in growth factors.

## Introduction

Since Marx first established a connection between the use of bisphosphonates for malignant bone diseases and the occurrence of necrotic bone in the oral cavity in 2003 (1), numerous studies have been published concerning the disease that soon became known as Bisphosphonate-Related Osteonecrosis of the Jaws (BRONJ) (2,3). The management of patients with BRONJ remains challenging because surgical and medical interventions may not eradicate this process. The goal of treatment of patients at risk of developing BRONJ, or for those who have active disease, is preservation of quality of life by controlling pain, managing infection, and preventing the development of new areas of necrosis (4). Not only the pathogenesis is still not fully understood, but also the treatment of BRONJ remains problematic and the outcome is often unknown (5,6).

For these reasons, different authors justify the application of alternative treatments, including hyperbaric oxygen (7), fluorescence-guided bone curettage (8), low intensity laser (9) and local flaps using buccal fat pad (10), but with no conclusive results.

The subcutaneous administration of teriparatide hormone (rhPTH1–34) (11,12) and the surgical application of plasma rich in growth factors (PRF) (13,14) in the surgical site constitute two new alternative forms of BRONJ treatment that should be evaluated.

The aim of this study is to describe and compare the evolution of BRONJ’s patients treated with plasma rich in growth factors (Group A) or teriparatide (Group B).

## Material and Methods

- Patients

The study was carried out with BRONJ patients belonging to a single hospital (reference for a population of 1.100.000 inhabitants) diagnosed and/or treated between January 2010 and December 2012. All patients must meet the following criteria: recurrent BRONJ after application of the protocol treatment proposed by AAOMS (15), impossibility of recommended surgery in stage 3 of classical classification (16) of BRONJ by poor general condition of the patient, absence of diagnosed neoplastic disease and lack of jaw radiotherapy medical history. All the patients were treated with PRF (approximately 7 ml in each patient) or teriparatide (20 μg once daily for each patient).

All the patients included in Group A had recurrences after previous surgeries with adverse outcome. A single surgeon (LJ) performed all the surgeries and applied the PRF in these patients. Post-surgical antibiotherapy was prescribed to all patients with amoxicilin/clavulanic acid (4 g/day) during 15 days. The patients were checked every 15 days during the following three months.

On the other hand, the group B consisted by those patients who had recurrences after a previous surgery and/or whose condition was generally impossible for the application of a new surgery. All the patients treated with teriparatide were previously evaluated by a specialist of the Bone and Mineral Metabolism Unit (CG), ruling out the presence of hypercalcaemia or unexplained eleva-tion of alkaline phosphatase.

- Types of treatment

A. Plasma rich in growth factors 

In order to obtain this plasma, it was used Vivostat PRF® system. Every patient was operated with general anesthesia or with local anesthesia and conscious sedation. During the surgery, 120 ml of blood were extracted. The Vivostat PRF® system is an automated, closed and sterile system that allows obtaining a platelet concentration ten times higher than the basal level and a fibrin concentration around 18.1 ng/ml.

**Figure 1:**
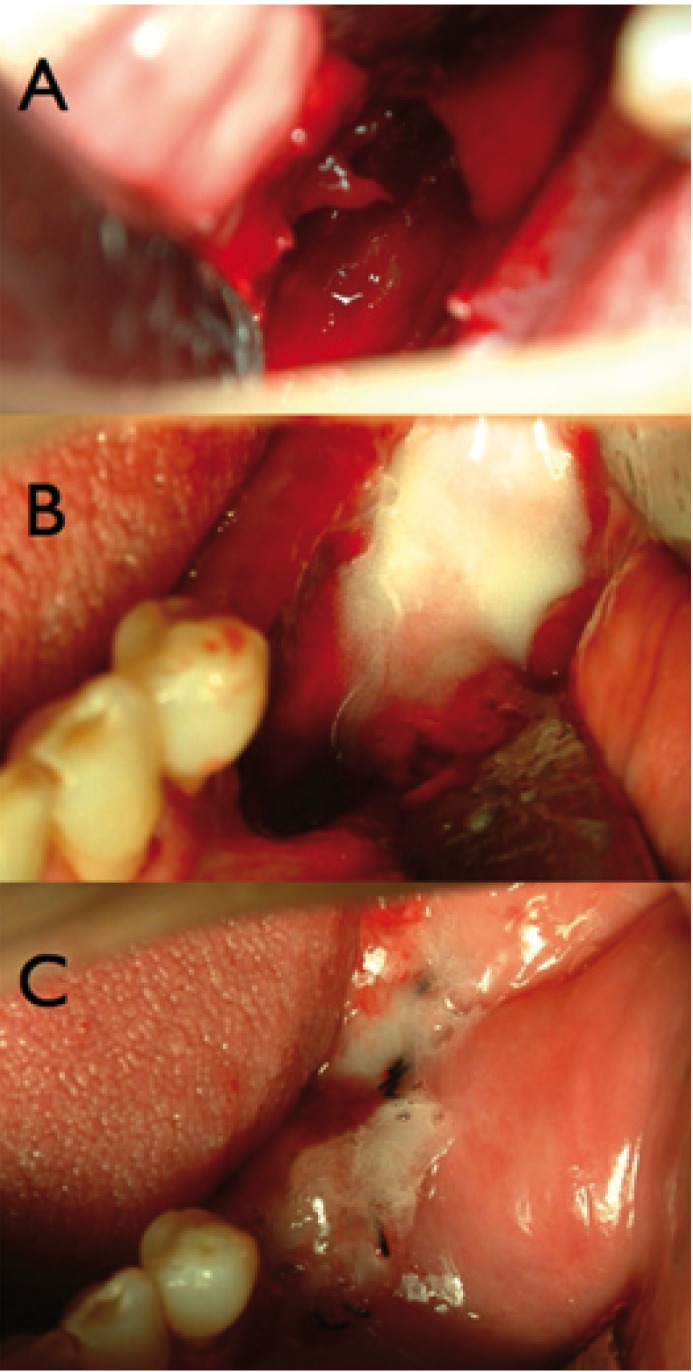
Case A2. Mandibular bone defect after sequestrectomy (A) filled with PRF (B). The mucosal wound is also covered with PRF (C).

Before the application of plasma with a pen sprayer, the bone sequestrations were removed and curettage was performed in the bone tissue until clear bleeding appeared from the subjacent bone (Fig. 1). The bone cavity was filled with the plasma obtained. A thorough hermetic close of the overlying oral mucosa was made with resorbable suture of 4/0 polyglycolic acid without tension in all the cases. Over the suture, a new layer of the product was also sprayed. The number of patients with this treatment was 5. For each patient were used about 7 ml of plasma rich in growth factors.

B. Teriparatide administration

Teriparatide (Forsteo®, 20 μg once daily by self-administered subcutaneous injection) was initiated at the baseline visit, and women attended monthly follow-up visits up to 6 months after discontinuing the teriparatide treatment. The maximum time of teriparatide administration was restricted to 10 months.

## Results

- Plasma rich in growth factors 

**Figure 2:**
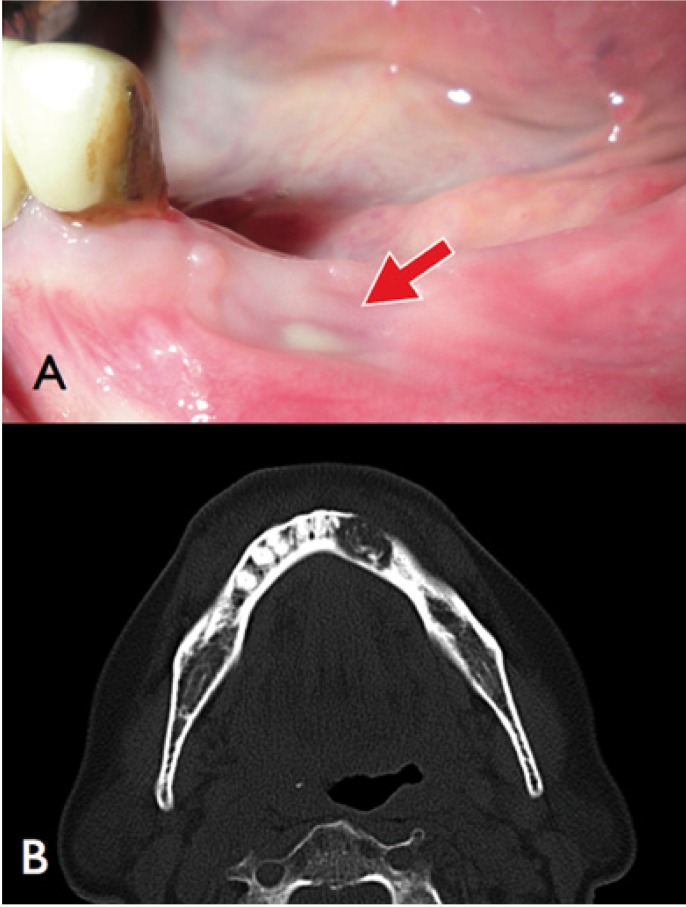
Case A4. Mandibular BRONJ with purulent sinus tract highlighted with an arrow (A). Computed tomography (B) shows the osteolytic pattern affecting the incisive mandibular area.

 Table 1 shows the main characteristics of the five patients belonging to the group with this treatment. The mean age was 72,8 years. All of them were women treated with oral bisphosphonates for osteoporosis. The drug most commonly used was the alendronate (4 cases). In four of the patients the exposed bone was located in the mandible. Three patients had a history of tooth extraction before the development of BRONJ and four patients had been previously treated with surgery in our hospital without success. In one patient, the bone exposure was located in the mandibular incisive region (Fig. 2) and had received no surgical treatment before application of PRF (Vivostat®). This patient not only had a previous history of multiple spontaneous expulsions of sequestered bone, but also the presence of active drainage at the time of diagnosis. The recovery of the disease was observed in all patients, with a complete resolution of the symptoms including the bone exposure. The average control time was 20 months (range: 12 to 24 months).

**Table 1:**
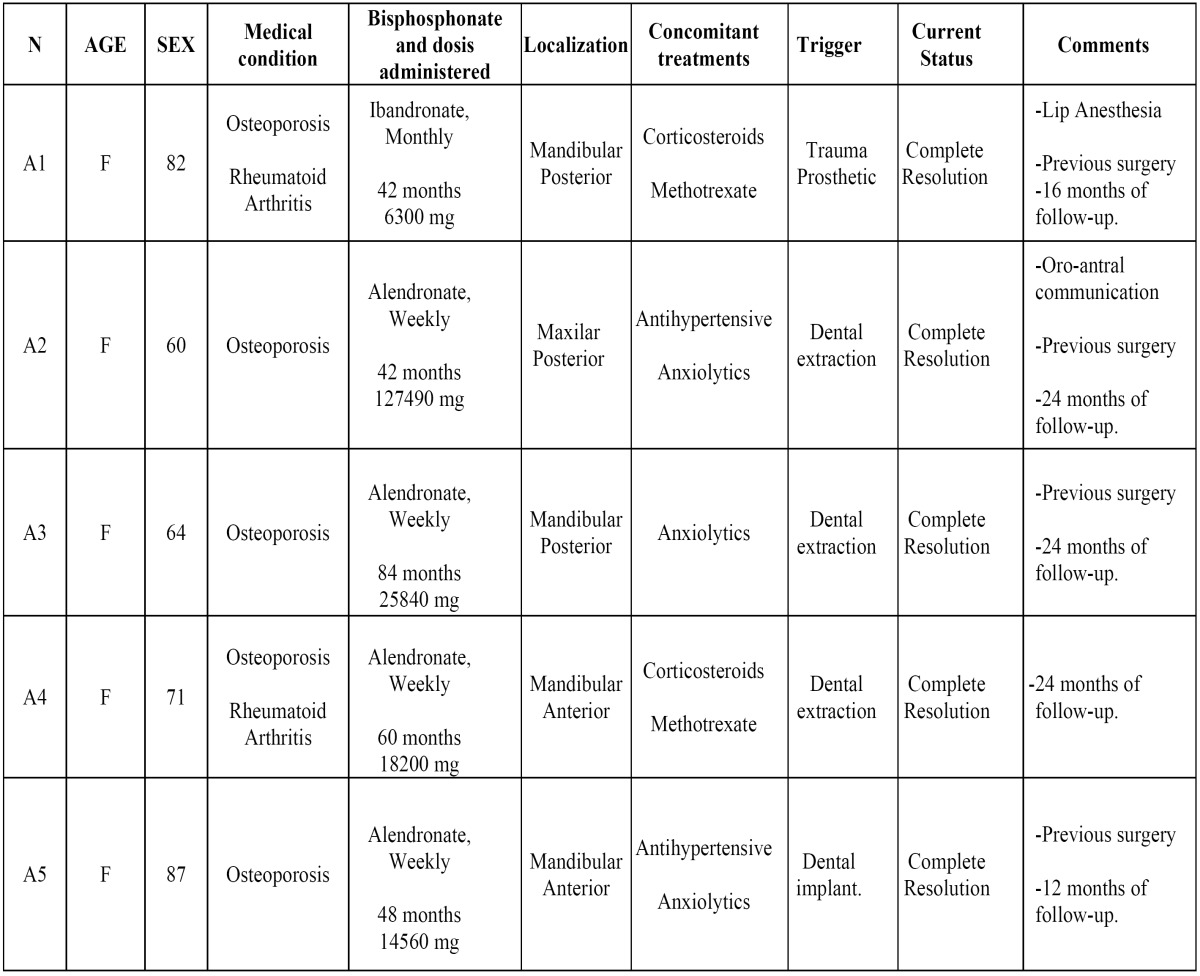
Summary of the patients treated with plasma rich in grow factors (PRF).

- Teriparatide

**Figure 3:**
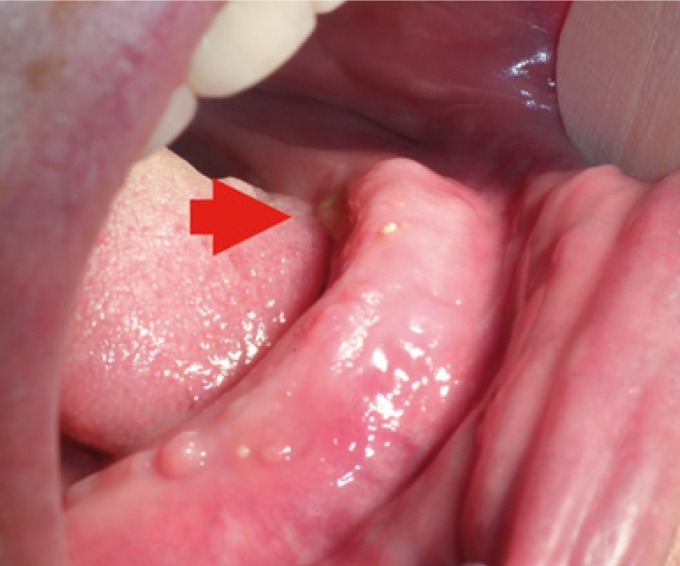
Case B3. Asymptomatic bone exposure after 7 months of treatment with teriparatide.

The main information from group B of patients is resumed in  Table 2. All of them were also women, with a mean age of 73.5 years. All the BRONJ were located in the mandible. Two patients had suffered a tooth extraction before the presentation of the BRONJ and had been subjected to sequestrectomy unsuccessfully in our service. Because of their general condition (severe neurodegenerative disease) the remaining two patients were excluded to any kind of surgical intervention. During the treatment, all patients were clinically and biochemically evaluated by the Bone and Mineral Metabolism Service. In the two patients without previous surgery, pain and infection were controlled (Fig. 3), but remained the bone exposure.

**Table 2:**
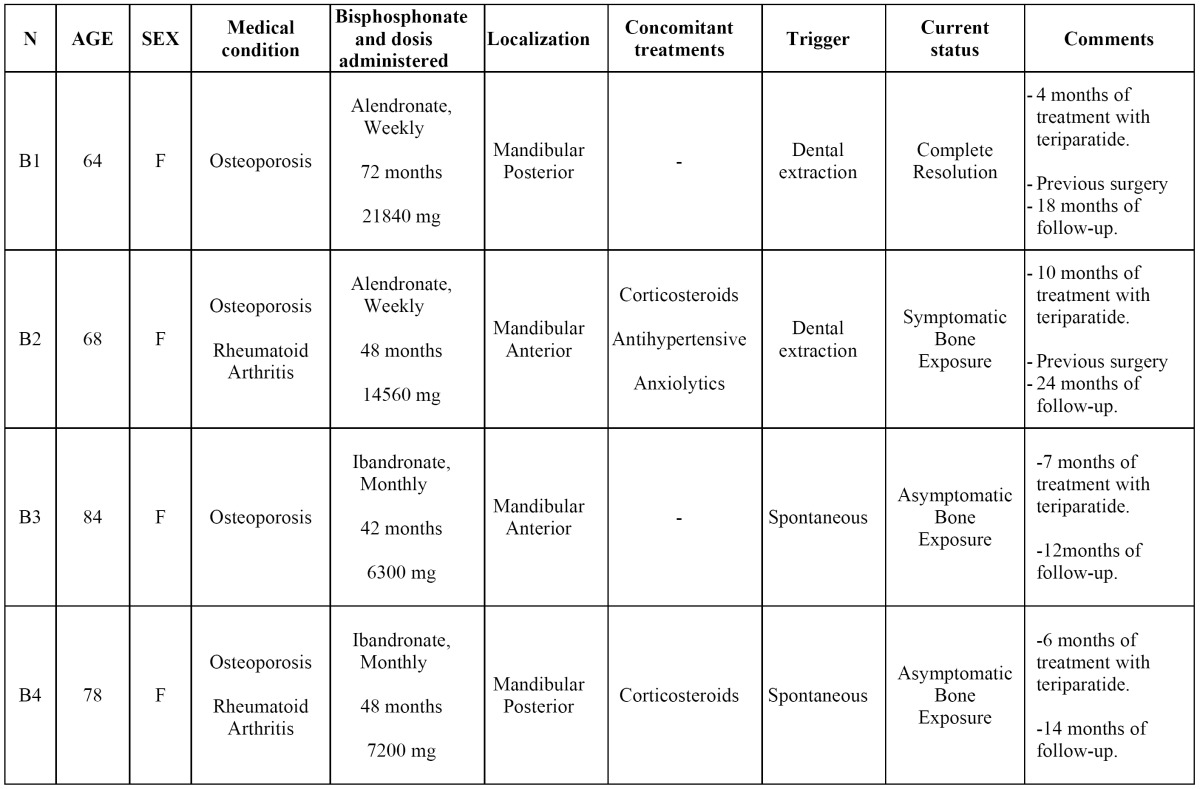
Main characteristics of the patients treated with teriparatide.

Regarding to the two patients with clinical history of recurrence after previous surgeries different outcomes were observed. One of them did not show any improvement with persistent symptomatic bone exposure after ten months of drug administration. Finally, the last patient developed a favorable outcome with complete resolution of the bone exposure after the removal of an additional bone sequestrum and associating a negative adherence to the teriparatide treatment abducting psychological problems. The teriparatide administration ranged from 4 to 10 months with an average control time of 17 months (range: 12 to 24 months).

## Discussion

Dohan Ehrenfest *et al.* (17) divided the platelet concentrates in four different types according to their leucocytes and fibrin content. In our study, fibrin rich in growing factors was used as filling material, using the technology of Vivostat PRF® system, with good results and long control time. In 2007 some observational studies were published about a reduced number of BRONJ patients who had been treated with PRF with irregular results (18-20). Recently, Bocanegra-Pérez *et al.* (13) published a series of 8 patients treated with platelet-rich plasma and leucocytes (Smart PReP®) showing an improvement of their lesions after a month with the treatment and also without bone exposure after 14 months. According to these authors, at least 2 of the 19 cases published with this treatment did not show any improvement, but the type of product employed is not uniform and the control time is often too short (less than 6 months). Very recently, Mozzati M *et al.* (14) have published an interesting essay that claims a 100% of success (complete healing) in 32 patients with chemical osteonecrosis, 24 of them in the mandible. Control time in the study was around 48-50 months. These authors used the technique described by Anitua, which also documented a BRONJ case treated successfully with PRF (21). Our observations show satisfactory results too although we had less cases and we used PRF. Moreover, it is important to note not only four of our patients treated this way had had been previously treated with surgery without success, but also three of the patients successfully treated with PRF wore more than one year of evolution of their BRONJ.

Lately, parathyroid human hormone (PTH) has been approved for the treatment of osteoporosis in two different presentations, 1-34 active fraction (Teriparatida, Forsteo®) and intact molecule (PTH, Protact®) identic to the natural complete hormone (and 84 aminoacid polypeptide). Teriparatide, recombinant human N-terminal fragment of parathyroid hormone, is a bone anabolic agent shown to increase bone mass and strength and reduces the incidence of vertebral and non-vertebral fractures in post-menopausal women with osteoporosis. The European Forsteo Observational Study (EFOS) was a prospective observational cohort study of women treated with teriparatide in eight western European countries. The primary objective of this study was to determine the incidence of clinical vertebral and non-vertebral fractures in post-menopausal women with osteoporosis treated with teriparatide for 18 months, with a post-treatment follow-up of 18 months. The results for the full cohort pooled across countries have recently been published (22). Recommended dose is 20 μg once a day subcutaneously into the thigh or abdomen during a maximum of 18 months. At this moment, despite its high cost (more than 5000 euros per year), it does not show better results than the regular alendronate treatment (23).

In October 2010, a prestigious international journal published a randomized, unicentric, double-blind study in which were compared the effects on the alveolar process of 40 patients with severe periodontal disease treated with 20 μg of teriparatide versus placebo during 6 weeks. After one year of clinical monitoring, the drug efficacy was proved (24). In the same number of this journal, other authors (11) documented an interesting case of an 88 year-old woman with an osteonecrosis caused by oral bisphosphonates with 12 months of evolution who had had no response to the regular treatment, including curettage. After starting with a 20-μg/day teriparatide treatment, clinical and radiographic resolution was shown at the 8th week of treatment.

More recently, Kwon *et al.* (25) published a work about 6 patients with osteonecrosis caused by oral bisphosphonates who were treated with teriparatide and showed the spontaneuos recovery of one of the patients after three months of treatment. The other 5 would also have a total recovery after practising surgery and antibiotherapy in a period not longer than three months. In this work, the only resolved case without another type of treatment but teriparatide was located in the upper jaw (the rest in the mandible).

In March 2012, Narváez *et al.* (26) reviewed all the cases published about BRONJ treated with teriparatide. They concluded that there were only six cases published (excluding the serie from Kwon *et al.*) (25) regarding osteonecrosis related to oral bisphosphonates (mostly alendronate). All of them presented a clinical and radiographic recovery (27-30). Teriparatide was given during 4-10 months. In 4 of the reviewed cases the factor that triggered the osteonecrosis was a dental extraction while in 2 cases was the placement of an implant. In their study, Narvaez *et al.* (26) give evidence of a 79 year-old patient with reumatoid arthritis with no previous surgery who did not show a resolution after being treated for 8 months with teriparatide.

In summary, 13 patients have been reviewed in the literature with oral bisphosphonate osteonecrosis who received subcutaneous teriparatide as treatment. In 12 of them clinical and radiologic recovery is referred and just in one the evolution could not be confirmed. In some cases, the osteonecrosis recovery was reached only with exclusive teriparatide treatment while in other ones it was necessary to combine bone anabolizant factor with antibiotics and surgery. According to this, it seems that the use of teriparatide as BRONJ treatment is still limited and that it has a low scientific evidence level.

It is paramount not to ignore the use of teriparatide is contraindicated in patients with metastatic cancer because of its participation in the promotion of metastasis and, furthermore, according to the FDA, its administration time may not be longer than 18 months, in relation to a possible osteosarcoma development (noticed in mice).

Our series details the less favorable results about the use of teriparatide in BRONJ reported in the literature; however, the patient choice was not randomized. We included two patients in which the protocol treatment proposed by AAOMS (15) had failed. One of those, with reumatoid arthritis treated concurrently with corticoids and methotrexate, did not present an improvement after 10 months of teriparatide treatment. The other patient, without concurrent diseases, a complete resolution was shown after practicing a new surgery after ending teriparatide treatment. Two people whose bad general condition did not indicate surgery formed the other patient group included in our study. In these two patients a total recovery of the BRONJ was neither reached after 7 and 6 months of teriparatide.

In conclusion, in our series the plasma rich in growth factors showed better results than the teriparatide in the treatment of recurrent BRONJ.
